# Retinal Changes before and after Silicone Oil Removal in Eyes with Rhegmatogenous Retinal Detachment Using Swept-Source Optical Coherence Tomography

**DOI:** 10.3390/jcm10225436

**Published:** 2021-11-21

**Authors:** Jungwook Lee, Heeyoon Cho, Minho Kang, Rimkyung Hong, Mincheol Seong, Yongun Shin

**Affiliations:** Department of Ophthalmology, College of Medicine, Hanyang University, Seoul 04763, Korea; mangoul@naver.com (J.L.); hycho@hanyang.ac.kr (H.C.); bsdoc@hanyang.ac.kr (M.K.); flarud1201@hanmail.net (R.H.)

**Keywords:** retinal thickness, rhegmatogenous retinal detachment, silicone oil tamponade, silicone oil removal, swept-source optical coherence tomography, OCT angiography

## Abstract

This study aimed to evaluate and compare the retinal and choroidal thickness and vessel density (VD) changes between silicone oil (SO) tamponade and after SO removal using swept-source optical coherence tomography (SS-OCT) and OCT angiography (OCTA). Thirty patients who underwent pars plana vitrectomy for retinal detachment (RD) with SO tamponade were included. SS-OCT and OCTA were conducted before RD surgery, during SO tamponade, and after SO removal. A 3-dimensional volumetric wide scan protocol was used for the analysis. The segmented retina, choroidal thickness map, and peripapillary thickness were then measured. For the OCTA analysis, 4.5 × 4.5 mm scans were used. Superficial and deep capillary plexus VDs in unaffected fellow eyes and eyes after SO removal were compared. During the SO tamponade period, the thickness of the parafoveal total retina, ganglion cell-inner plexiform layer, and peripapillary retinal nerve fiber layer (ppRNFL) were significantly thinner than those of unaffected fellow eyes (*p* < 0.05). The parafoveal layer thickness thinning recovered up to three to six months after SO removal. Moreover, six months after SO removal, the parafoveal thickness was not significantly different compared to that of unaffected fellow eyes (*p* > 0.05). However, the ppRNFL thickness was significantly decreased during SO tamponade and remained unrecovered six months after SO removal. There was no significant difference in the VD on the OCTA. Thus, SO tamponade and removal for RD resulted in a change in the retinal and peripapillary thickness. This may be due to the mechanical pressure effect of SO.

## 1. Introduction

Silicone oil (SO) tamponade is commonly used as a long-term endotamponade to manage various retinal diseases, such as proliferative vitreoretinopathy, complicated tractional or rhegmatogenous retinal detachment (rRD) with a giant retinal tear, and severe traumatic retinal detachment (RD). However, there are many reports about anterior and posterior ocular complications associated with SO, including band keratopathy, secondary glaucoma, cataract, increased intraocular pressure, and SO emulsification [1,2,3,4].

Another complication of SO tamponade is unexplained visual impairment [2]. Several studies investigated the effect of SO tamponade on the retinal layer, using spectral-domain optical coherence tomography (SD-OCT). SD-OCT studies suggested that SO tamponade causes thickness changes in the macular inner retinal layer such as in the retinal nerve fiber layer (RNFL) and ganglion cell-inner plexiform layer (GC-IPL). Ultimately, these changes may be related to visual impairment [1,3,4,5]. However, the exact mechanism remains unknown. 

Most previous SO-related studies using SD-OCT assessed only the retinal thickness changes in a part of the macular area due to a limited scan protocol. Swept-source OCT (SS-OCT) is the latest commercially available OCT platform that significantly improves image acquisition and resolution compared with SD-OCT [6]. In addition, SS-OCT uses a long wavelength that can penetrate deeper tissue well; thus, it is well-suited for choroidal imaging and imaging of eyes with media opacity. However, no study has been performed on the changes in the retinal layer during SO tamponade using SS-OCT. Nevertheless, the usage of a wide scan protocol of SS-OCT enables the measurement of the thickness of the retina and choroid in a wide area, including the macula and peripapillary area, simultaneously with a single scan. 

Recently, commercially available OCT angiography (OCTA) was introduced in the clinical setting. OCTA can visualize layer-specific retinal vasculatures, such as the superficial capillary plexus (SCP), deep capillary plexus (DCP), and choriocapillaris (CCP), noninvasively [7]. The non-invasiveness of OCTA enables the evaluation of retinal perfusion status repeatedly and easily. Although OCTA has several limitations, such as having a narrow scan area and producing artifacts, it is used by researchers to investigate the changes in the retinal perfusion status in various retinal diseases [8,9].

The purpose of this study was to compare various segmented retinal layer thicknesses in the macula and peripapillary areas before and after SO removal using wide-scan imaging with SS-OCT and to determine how SO tamponade affected these thickness changes. In addition, we evaluated the vessel density (VD) after SO tamponade using SS-OCTA.

## 2. Materials and Methods

### 2.1. Study Design and Population

We reviewed the medical records of 30 patients who underwent a 23-gauge pars plana vitrectomy with SO tamponade for the repair of rRD between October 2016 and November 2019 at Hanyang University Guri Hospital, Korea. This study was approved by the Institutional Review Board (IRB) of Hanyang University Guri Hospital and was conducted in accordance with the tenets of the Declaration of Helsinki (IRB no. 2019-12-031-003). The IRB approved a waiver of informed consent due to the retrospective study design. 

The patients were consecutively selected according to the following inclusion criteria: 40 years of age or older, primary rRD in the affected eye, a healthy fellow eye, and the absence of a history of previous retinal surgery in either eye. The exclusion criteria were as follows: cystoid macular edema on OCT in the SO tamponade state, a high refractive error (spherical equivalent of more than ±6 D), a long axial length (≥26.0 mm), an axial length difference of more than 0.3 mm between the eyes, eyes with any retinal pathology such as diabetic retinopathy, retinal vein occlusion, epiretinal membrane, glaucoma, and optic nerve abnormalities, and poor OCT image quality. 

Standard 23-gauge pars plana vitrectomy and SO tamponade were performed in all patients. Surgeries were performed by either of the two surgeons, H.C. and Y.U.S. RD was repaired in a standard fashion after vitreous removal and shaving of the periphery. The silicone oil used in this study was Oxane 5700 (Bausch & Lomb Inc., Waterford, Ireland). Cataract surgery was also performed in patients who had a high-grade cataract (over N3 or P3 on Lens Opacities Classification System III (LOCS III)) and in those who were over 60 years old. In all cases, the retina was completely attached at the end of the procedure. After surgery, the patients were asked to remain in a prone position for one or two weeks. 

### 2.2. Examination Procedures

Before and after surgery, all patients underwent comprehensive ophthalmological examinations, including the evaluation of best corrected visual acuity (BCVA), slit lamp examination, dilated fundus examination, intraocular pressure (IOP) measurement using a non-contact tonometer, automated keratometry, axial length measurement using IOL Master 500 (Carl Zeiss Meditec AG, Jena, Germany), and SS-OCT measurements (DRI OCT-1 Atlantis; Topcon Corporation, Tokyo, Japan). Trained examiners performed all examinations, and images were taken before RD surgery, one week after SO tamponade, before SO removal, and one week, three months, and six months after SO removal. The unaffected fellow eye was used in analyses for comparison with post-operative eyes. Cases in which the axial length of the preoperative fellow eye differed from that of the affected eye after retinal reattachment (±10%) were excluded. 

**Figure 1:**
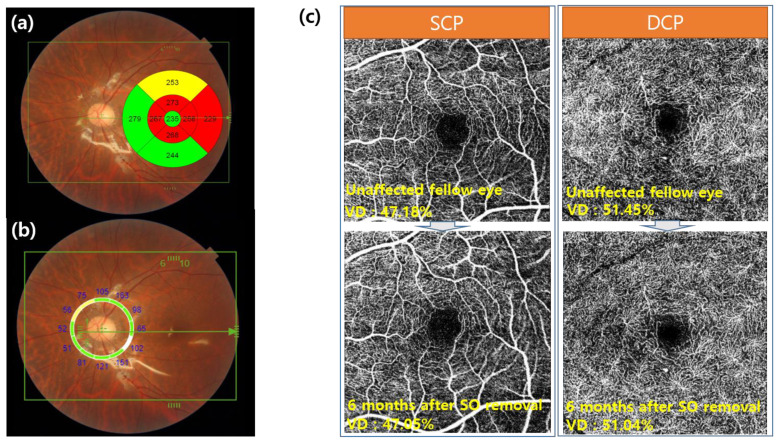
Representative images of this study. (**a**) ETDRS retinal thickness and (**b**) peripapillary retinal nerve fiber layer (ppRNFL) thickness were measured simultaneously using the SS-OCT 3D volumetric wide scan mode (12 × 9 mm) in the silicone oil tamponade state. (**a**) ETDRS retinal thickness can be created to illustrate the thickness deviation (from normative data) within a 6 × 6 mm ETDRS map, using a color-coded scale. The ETDRS map is centered on the fovea. Yellow pixels (representing a thickness <5% of the normative level) or red pixels (representing a thickness <1%), indicating retinal thinning, were considered abnormal in our analyses. It can be seen that the thickness of the parafovea region (inner circle) is relatively thin. (**c**) Vessel density was taken from unaffected fellow eyes and eyes at six months after SO removal using SS-OCT angiography (4.5 × 4.5 mm). There are no significant changes compared with the unaffected fellow eye. SCP: superficial capillary plexus, DCP: deep capillary plexus, SO: silicone oil, VD: vessel density.

SS-OCT used in this study had a faster scanning speed of 100,000 A-scans/s and longer wavelength light source than SD-OCT, which enabled visualization of the microstructures beneath the retinal pigment epithelium and provided better images even with media opacity. In particular, the center wavelength of the probe beam was 1060 nm. The scan protocol involved a 3D volumetric wide scan mode covering 9 × 12 mm, which enabled the simultaneous capture of the macular and optic discs. The early treatment diabetic retinopathy study (ETDRS) subfields were divided into two distinct subgroups: the foveal 1 mm group and the parafoveal 3 mm group (Figure 1).

The automated retinal segmentation technique built on SS-OCT was used to distinguish each retinal layer and calculate its thickness automatically [10]. Hence, the total retina, RNFL, GC-IPL, peripapillary RNFL, and choroidal thickness were measured using the built-in software. Two retinal specialists (Y.U.S. and H.C.) manually reviewed and corrected all OCT segmentation errors. The data obtained by calibration by two people were averaged and analyzed.

OCTA was performed to determine the relationship between the change in the thickness of the segmented retinal layers and the perfusion state. OCTA was performed before surgery and six months after SO removal. All eyes were scanned using a 4.5 × 4.5 mm protocol centered on the fovea. These scan images were automatically segmented by the software into en-face slabs: (1) the SCP: from 2.6 μm below the internal limiting membrane to 15.6 μm below the interface of the inner plexiform layer and inner nuclear layer (IPL/INL) and (2) the DCP: from 15.6 μm below the IPL/INL to 70.2 μm below the IPL/INL [11]. The “Auto Local Threshold” of ImageJ Landini G., version 1.5, was used to separate the retinal microvasculature from the background noise. The “Phansalkar method” with a radius of 15 was used for the binarization algorithm. The VD value was defined as the proportion of an angiography signal in the whole 4.5 × 4.5 mm macular area [12]. In addition, we used the SCP to evaluate the FAZ area. The area of the foveal avascular zone (FAZ) was determined via manual delimitation of the superficial retinal vascular layers from OCTA with image6.net software [13].

The main outcomes measured in this study included changes in the thickness of the total retina, inner retinal layer (RNFL, GC-IPL), choroid in the macular area, peripapillary RNFL, perfused macular VD, and FAZ between SO tamponade and after SO removal.

### 2.3. Statistical Analyses

Statistical analyses were performed using the SPSS software, version 22.0 (SPSS Inc., Chicago, IL, USA). The mean macular retina, choroidal layer, and peripapillary RNFL thicknesses in unaffected fellow eyes were compared with those before and six months after SO removal. Each measured layer was evaluated before SO removal and one week, three months, and six months after SO removal via the Linear mixed model (LMM) test to account for inter-individual differences. A correlation analysis between the final visual acuity and changes in retinal thickness was performed. Finally, a univariate correlation analysis and Spearman correlation analysis were used to analyze the relationship between final visual acuity and baseline characteristics. 

## 3. Results

### 3.1. Demographic Data

In this study, we retrospectively included 40 eyes of 40 patients who underwent the repair of rRD with SO tamponade. We then applied the exclusion criteria. As a result, 30 eyes of 30 patients were finally analyzed. The mean age of the enrolled patients was 58.53 ± 9.28 years (range, 41–71 years; 17 men and 13 women). Twenty-six eyes had macular-off RD, and four eyes had macular-on RD at diagnosis. The mean period of SO tamponade was 3.14 ± 1.4 months (range, 2–7 months; ≤3 months, 19 eyes; >3 months, 11 eyes). The mean preoperative BCVA was 0.76 ± 0.85 logMAR at diagnosis and 0.16 ± 0.27 logMAR six months after SO removal (*p* = 0.01). The mean IOP was 14.21 ± 3.09 mmHg before RD surgery and 14.85 ± 2.47 mmHg six months after SO removal. These were not significantly different (*p* = 0.45). Furthermore, the mean spherical equivalent was −2.45 ± 3.04 at diagnosis and −2.39 ± 3.14 six months after SO removal (*p* = 0.51). In the macular-off cases, the mean axial length was 24.98 ± 1.90 mm in the affected eyes after reattachment and 24.54 ± 1.36 mm in the unaffected fellow eyes (*p* = 0.072). All patients underwent surgery without complications. There was no recurrence of RD until the last follow-up day. Detailed demographic data are presented in Table 1.

### 3.2. Changes in Retinal, Choroidal, and Peripapillary Thickness after SO Removal 

**Figure 2:**
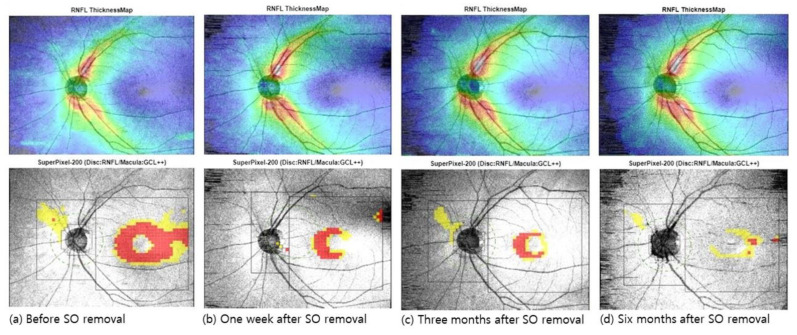
The upper row is the retinal nerve fiber layer (RNFL) thickness map, while the lower row is the deviation map for the disc RNFL and macular ganglion cell layer (GCL) thickness (**a**) before SO removal, (**b**) one week after SO removal, (**c**) three months after SO removal, and (**d**) six months after SO removal. Before SO removal, the parafoveal GCL thickness was decreased. Contrastingly, after SO removal, the thickness was gradually recovered.

**Figure 3:**
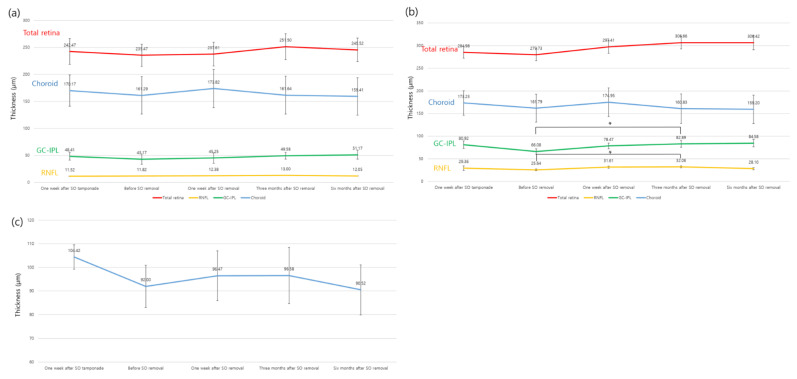
The segmented retinal layer thickness changes from one week after SO tamponade to six months after SO removal. (**a**), fovea, (**b**), parafovea, (**c**), peripapillary retinal nerve fiber layer (RNFL). SO: silicone oil, GC-IPL: ganglion cell-inner plexiform layer. Bar means 95% confidence interval. * Statistically significant difference (*p* < 0.05) by post hoc analysis between before SO removal and three months after SO tamponade.

Table 2 and Figure 2 and Figure 3 show the retinal and choroidal layer thicknesses in the foveal and parafoveal areas of unaffected fellow eyes one week after SO tamponade; before SO removal; and one week, three months, and six months after SO removal. Up to three months after SO removal, all segmented retinal layers, which included the total retina, RNFL, and GC-IPL, in the macula were gradually thickened. However, six months after SO removal, the GC-IPL showed an increase in thickness, while others did not show additional recovery. None of the segmented macular retinal layer thicknesses at six months after SO removal differed statistically from those of unaffected fellow eyes (Table 2 and Figure 3).

The thickness of the total retina, RNFL, and GC-IPL in the macular area before SO removal was thinner than that of unaffected fellow eyes. In particular, only the parafoveal GC-IPL and total retina showed statistically significant thinning (*p*-value: total retina, 0.007; GC-IPL, 0.005). Before SO removal, the choroidal thickness was lower (fovea, 161.29 ± 45.48 µm; parafovea, 161.79 ± 44.16 µm) than that of unaffected fellow eyes (184.29 ± 50.21 µm and 180.47 ± 41.89 µm in the fovea and parafovea, respectively); however, there was no statistical difference. At one week, three months, and six months after SO removal, the foveal and parafoveal choroidal thicknesses were 173.82 ± 48.52 µm and 174.95 ± 43.25 µm, 161.64 ± 51.59 µm and 160.83 ± 46.93 µm, and 159.41 ± 52.74 µm and 159.20 ± 47.55 µm, respectively. Approximately six months after SO removal, there was a borderline statistical difference from that of unaffected fellow eyes (*p*-value; fovea, 0.053; parafovea, 0.06) (Table 2, Figure 3).

The peripapillary RNFL thickness was significantly lower during the SO tamponade period than that of unaffected fellow eyes (unaffected fellow eyes, 110.65 ± 16.21 µm, before SO removal, 92.0 ± 19.68 µm, *p* = 0.002). The thickness of the peripapillary RNFL increased up to one week and then three months after SO removal (one week, 96.47 ± 18.54 µm; three months, 96.58 ± 17.46 µm). However, after that, the thickness decreased again (six months, 90.52 ± 15.35 µm). When comparing unaffected fellow eyes and eyes at six months after SO removal, there was a statistically significant difference in thickness (p = 0.003) (Table 2 and Figure 3).

We evaluated whether changes in retinal and choroidal thickness were statistically significant during the follow-up period (before the SO removal period at baseline) using LMM (Table 3). In the foveal region, there were no statistically significant changes in any of the segmented layers (*p*-value: total retina, 0.426; RNFL, 0.988; GC-IPL, 0.226; choroid, 0.920). In the parafovea region, there were statistically significant changes in the total retina, RNFL, and GC-IPL (*p*-value: total retina, 0.049; RNFL, 0.009; GC-IPL, 0.003; choroid, 0.865). The peripapillary RNFL thickness increased significantly at three months but was not significant at six months after SO removal (*p* = 0.80). 

To determine the effect of SO tamponade duration on the recovery of parafoveal retinal thickness after SO removal, the period of SO tamponade was divided into two subgroups based on three months: ≤three months and >three months (Table 4). In both subgroups, the difference in retinal thickness between unaffected fellow eyes and eyes at six months after SO removal was not statistically significant according to the SO tamponade period (*p* > 0.05). 

### 3.3. Correlation between Visual Acuity and Thickness Changes

The final logMAR BCVA (at 6 months after SO removal) was 0.16 ± 0.27, which showed a clear improvement compared with that before RD surgery. However, there was no significant correlation between the final BCVA and all baseline characteristics except macular status (on/off) (spearman correlation, *p* = 0.022). Whether the macula was attached preoperatively was significantly correlated with visual acuity.

### 3.4. Changes of the VD on OCTA 

The average VDs of the SCP and DCP in unaffected fellow eyes were 47.18 ± 2.87% and 51.45 ± 2.69%, respectively (Table 1). At six months after SO removal, the average VDs decreased slightly to 47.05 ± 2.91% and 51.04 ± 3.14%, respectively; however, there was no statistically significant difference in both the SCP and DCP (*p*-value: SCP, 0.862; DCP, 0.747). The mean FAZ area of unaffected fellow eyes and eyes at six months after SO removal was 352.81 ± 191 µm^2^ and 323.18 ± 250 µm^2^, respectively, without any statistically significant difference between the two groups (*p* = 0.13).

## 4. Discussion

In this study, we evaluated changes in retinal and choroidal thickness before and after SO removal using SS-OCT and OCTA. During SO tamponade, the overall retinal thickness in the parafoveal area was lower than that in unaffected fellow eyes; however, the thickness recovered up to three to six months after SO removal. Among the segmented retinal layers, parafoveal GC-IPL showed statistically significant changes. Meanwhile, macular choroidal thickness did not show a significant change between before and after SO removal. The peripapillary RNFL was significantly thinner after SO tamponade than that of unaffected fellow eyes, and this change did not recover after SO removal. OCTA variables (SCP, DCP, and FAZ) did not show significant differences between unaffected fellow eyes and eyes at six months after SO removal.

SO is a chemical compound and a polymer of polydimethylsiloxane. It forms a spherical bubble inside the vitreous cavity and exerts force on the eye as a result of buoyancy, volume displacement, and surface tension of the water-oil boundary. Due to these physical properties, it has been used in retinal surgery requiring long-term endotamponade. However, previous studies reported that it may cause some adverse effects on the retina [14,15,16].

Our study found that the retinal thickness of the parafovea in SO tamponade was lower than that of unaffected fellow eyes. The lower retinal thickness may be due to the effect of the SO tamponade effect or retinal damage induced by macular-off RD. Our study has a limitation in the interpretation of the SO effect because most enrolled patients had macula-off RD. However, Kim et al. reported that a significant decrease in outer retinal layers, particularly the outer nuclear layer and photoreceptor layer, was observed in patients with macula-off RD using SD-OCT for a follow-up of ≥6 months, while inner retinal thickness did not show any significant change [17]. Other experimental studies reported similar results [18]. Therefore, we assumed that the thinning of the inner retinal thickness was caused by SO tamponade rather than macular-off RD itself. Previous studies reported that the IOP affected the thickness of the retina and choroid [19,20]. However, in our study, there were only eight eyes with an elevated IOP during the follow-up period. Most of the patients’ eyes were controlled well with anti-glaucoma eye drops; thus, elevated IOP might not have affected the retinal thickness changes. There are many SD-OCT studies on retinal thickness changes related to SO tamponade, especially in the inner retina. Caramoy et al. reported that the ganglion cell and inner retinal layers subsequently became thinner after SO tamponade [21]. Furthermore, Takkar et al. reported that the thickness of the macular RNFL was decreased in eyes with SO tamponade for RD surgery [3]. In accordance with the two aforementioned studies, Purskhvanidze et al. reported that the inner retinal layer showed significant thinning in the fovea/parafovea after SO tamponade when compared with that after gas tamponade [4]. Moreover, there are also other SD-OCT studies related to retinal thickness changes after SO removal. Gilad et al. reported that SO tamponade caused a transient decrease in the central macular thickness mainly of the inner layers. However, the thickness seemed to recover after SO removal [22,23]. This finding was supported by the finding by Danielle et al., who divided the subjects into two groups depending on the retinal thickness (thicker vs. thinner group) during SO tamponade. They found that both groups showed thickness recovery after SO removal [24]. However, previous SD-OCT studies had limitations. The scan protocol used in those studies could cover only a limited area, such as the subfoveal or macular area. 

The SS-OCT protocol used in our study could cover the whole macular and peripapillary area and could construct ETDRS and peripapillary RNFL thickness maps simultaneously. As our results and previous studies suggested, SO tamponade could influence the retinal structure. Several hypotheses have been proposed regarding the cause of retinal structure changes after SO tamponade. Takkar et al. assumed that the retinal accumulation of potassium and subsequent neuronal degeneration might induce inner retinal thinning [3]. Sebastian et al. reported that retinal thinning induced by the mechanical pressure of SO could be a possible cause [25]. In addition, SO toxicity and dehydration were hypothesized as potential mechanisms of retinal thinning; however, this remains unproved to date [23].

**Figure 4:**
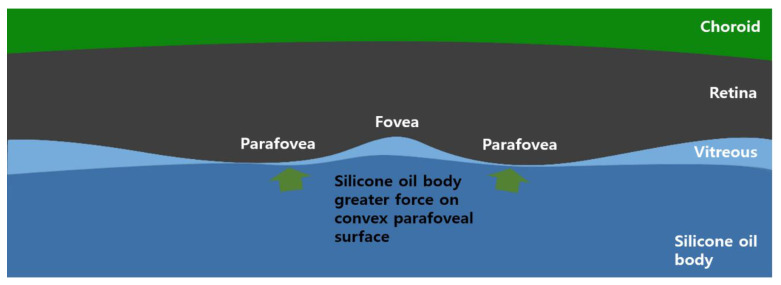
This image illustrates the macular area in the silicone oil tamponade state in the prone position. It can be assumed that a greater force can be applied on the parafoveal surface due to the convex curvature in the prone position.

We assumed that retinal thinning in the SO tamponade state was caused by two potential mechanisms: physical pressure conferred by the SO and disturbance of the retinal blood flow. As tamponade-inducing agents, buoyancy and surface tension are important physical properties of SO [26,27,28,29]. The specific gravity of SO is 0.971, and this provides a compression effect on the macula in the prone position. Our results showed a significant change in the inner retinal thickness in the parafoveal area in the SO tamponade state (Table 4). This may be due to the convex curvature of the parafoveal area, where a greater force can be applied in the prone position (Figure 4). 

The recovery pattern of retinal thickness after SO removal was analyzed using LMM (Table 3). The statistical significance of the recovery pattern was different for each layer, but it was observed mainly in the parafovea. Specifically, the parafoveal RNFL and GC-IPL thickness recovery were achieved at three months, and that of total retinal thickness was achieved over six months after SO removal by post hoc analysis. However, the parafoveal RNFL thickness did not show a statistical difference at six months after SO removal. There were some previous studies on retinal changes after SO removal [22,23]. Bae et al. reported that the macular microstructure changes after SO tamponade recovered mainly up to three months after SO removal [22]. In addition, Gilad et al. reported that central macular transient thinning was restored one month after SO removal [23]. However, these studies had limitations in terms of using SD-OCT and the relatively short-term follow-up period.

Previous SD-OCT studies reported a decrease in the subfoveal choroidal thickness after SO tamponade [30,31,32]. Our study did not show any significant thickness difference between before and after SO removal. Compared with the changes in retinal thickness, we assumed that the choroidal thickness was less affected by SO because the choroid was located in a relatively deep area, which was less likely to be mechanically affected. After SO removal, the choroidal thickness did not show a meaningful change compared to that before SO removal (LMM, *p* > 0.05). In this study, we analyzed the macular choroidal thickness map covering the whole macula using SS-OCT, which might generate more reliable data than prior studies. Nevertheless, further research is warranted to confirm the effect of SO on the choroid. 

The peripapillary RNFL was thinner after SO tamponade than that of unaffected fellow eyes. However, the thickness did not recover even six months after SO removal. There were several studies on the changes in the peripapillary RNFL thickness after SO tamponade [1,33]. Geber et al. reported that the peripapillary RNFL thickness was significantly greater compared with that of the fellow unoperated eye over a six-month period [1]. These authors postulated that there was a possibility of surgical manipulation (endolaser treatment, induction of posterior vitreous detachment), early postoperative retinal readjustment, and subclinical toxic effects of SO. In addition, Jurišić et al. also reported thickening of the peripapillary RNFL during SO tamponade, which was slightly reduced after SO removal [33]. In contrast, in our study, the peripapillary RNFL showed thinning after SO tamponade, but it did not recover after SO removal for up to six months. This change in peripapillary RNFL thickness was different from the reversible characteristics of the inner retina after SO removal. The reason for this remains unknown; however, recent OCTA studies from Lu et al. reported that peripapillary vessel density in eyes with macula-off was lower than that in unaffected fellow eyes three months after vitrectomy although they used silicone oil as a tamponade material in only eight eyes (25.8%) [34]. Both peripapillary blood flow disturbance and the SO effect may affect prolonged peripapillary thinning, but additional research is warranted.

Our study showed that there was no significant difference between the groups with SO tamponade duration less and more than three months on the recovery of parafoveal retinal thickness. The final BCVA had no significant association with inner retinal change or SO tamponade duration in this study. This result is similar to that of a previous study [23,35]. Furthermore, Karasu et al. reported that there was no statistically significant change in the macular thickness due to the SO tamponade period [35], and Rabina et al. reported that the degree of retinal thinning did not correlate significantly with postoperative final visual acuity [23]. However, Shalchi and Lee et al. reported that inner macular thinning after SO tamponade can be related to poor visual outcomes [5,36]. Various factors, such as the RD duration and surgical method, seem to influence the differences in these results [37].

We measured the FAZ area and VDs of the SCP and DCP using SS-OCTA to identify the relationship between retinal thickness and retinal blood flow. Previous SD-OCT studies reported that retinal thickness, especially that of the inner retina, decreases if macular perfusion decreases [38,39]. Our data showed that the FAZ area and VD were not statistically different between unaffected fellow eyes and eyes at six months after SO removal. Bonfiglio et al. reported that there was no difference in the FAZ in eyes with gas tamponade for rRD surgery compared to that in fellow eyes [40]. These findings were similar to ours. However, our study has limitations. OCTA was not performed in the SO tamponade state because of the low quality of the OCTA images. 

Based on our OCTA results, although this study could not measure the VD at the time of SO tamponade, SO did not affect the blood flow to the macular inner retina. Even if it did, the flow might have recovered after SO removal. If SO did not affect retinal blood flow, the decrease in the inner retinal thickness in SO tamponade was more influenced by the pressure effect. However, further research is required to confirm this mechanism. 

As mentioned previously, this study has limitations. First, it was a retrospective study with a small sample size. Second, this study did not include preoperative control data. In cases with macula-on RD, preoperative OCT data can serve as a control to determine the SO effect on the retina during the SO tamponade period. However, most enrolled patients in this study had macular-off RD. Eyes with macular-off RD might have retinal edema and poor fixation, which could lead to inaccurate measurements, thereby making it difficult to use as a preoperative control. Inevitably, we used the thickness of unaffected fellow eyes to identify the SO tamponade effect on the retina and excluded the cases with axial anisometropia to make similar conditions before macular-off RD. Third, the prone position maintenance period was not constant for each patient. Mechanical compression and IOP in the SO tamponade state could have more influence on the retina in the prone position than in the sitting position, which could have caused bias in this study [41]. Finally, OCTA data were not obtained during SO tamponade because the OCTA image quality was so poor due to the reflection of SO that the images could not be used for analysis. 

Nevertheless, to the best of our knowledge, this is the first study to evaluate the thickness of the retina, choroid, and optic disc simultaneously before and after SO removal using SS-OCT. Due to the superior resolution and high speed of SS-OCT, we evaluated a wide area of the retina and analyzed the thickness map of the entire macular area more accurately. Another strength of this study was the fact that we evaluated perfused VD using SS-OCTA after SO removal. A longer follow-up period after SO removal than in previous studies was also an additional strength of our study.

## 5. Conclusions

SO tamponade causes temporary thinning of the parafoveal retina, especially the inner retina, because of the potential effect of the mechanical pressure of SO on the retina, especially in the prone position. These retinal changes recovered after SO removal. After recovery, the inner retinal thickness is similar to that of unaffected fellow eyes. However, peripapillary RNFL thinning in the SO tamponade state remains constant even after SO removal. The perfused macular VD and FAZ may not be affected by SO. Further research is needed to determine which visual functions are affected by SO-induced thickness changes. 

## Figures and Tables

**Table 1:** Baseline clinical characteristics of patients undergoing vitrectomy with silicone oil tamponade due to rhegmatogenous retinal detachment.

Clinical Parameter		*p*-Value
Age (y)	58.53 ± 9.28	
Sex (male/female)	17/13	
Macular status (on/off)	4/26	
Phakic/Pseudophakic	9/21	
Mean duration of SO tamponade (months)		
≤3 months (*n* = 19)		
>3 months (*n* = 11)	3.14 ± 1.4 (2–7 months)	
BCVA (LogMAR)		0.01 *
preoperation/six months after SOR	0.76 ± 0.85/0.16 ± 0.27	
IOP (mmHg)		
preoperation/six months after SOR	14.21 ± 3.09/14.85 ± 2.47	0.45
Spherical equivalent refractive error (diopter)		
preoperation/six months after SOR	−2.45 ± 3.04/−2.39 ± 3.14	0.51
Axial length (mm)		
unaffected fellow eye	24.54 ± 1.36	
six months after SOR	24.98 ± 1.90	0.072
SCP vessel density (%)		
unaffected fellow eye/six months after SOR	47.18 ± 2.87/47.05 ± 2.91	0.862
DCP vessel density (%)		
unaffected fellow eye/six months after SOR	51.45 ± 2.69/51.04 ± 3.14	0.747
FAZ area (µm^2^)		
unaffected fellow eye/six months after SOR	352.81±191/323.18 ± 250	0.13

* Statistically significant difference (*p* < 0.05, paired *t*-test); BCVA, best corrected visual acuity; SOR, silicone oil removal; LogMAR, logarithm of the minimum angle of resolution; IOP, intraocular pressure; RD, retinal detachment; SO, silicone oil; SCP, superficial capillary plexus; DCP, deep capillary plexus; FAZ, foveal avascular zone.

**Table 2:** Retinal and choroidal thickness changes in the fovea and parafovea ETDRS subfields before and after silicone oil removal.

					Follow-Up Period			*p*-Value		
		Unaffected Fellow Eye	1 Week after SO Tamponade	Before SOR	1 Week after SOR	3 Months after SOR	6 Months after SOR	Before SOR vs. 1 Week after SOR	Before SOR vs. 3 Months after SOR	Unaffected Fellow Eye vs. 6 Months after SOR
Fovea (µm)	Total retina	251.82 ± 47.03	242.47 ± 39.97	235.47 ± 21.37	237.61 ± 21.50	251.50 ± 20.77	245.52 ± 20.98	0.75	0.078	0.48
	RNFL	14.00 ± 11.31	11.52 ± 8.61	11.82 ± 10.28	12.38 ± 13.01	13.00 ± 11.21	12.05 ± 10.98	0.92	0.45	0.63
	GC-IPL	49.82 ± 19.64	48.41 ± 18.64	43.17 ± 18.27	45.25 ± 20.11	49.58 ± 17.49	51.17 ± 19.67	0.69	0.09	0.327
	Choroid	184.29 ± 50.21	170.17 ± 57.67	161.29 ± 45.48	173.82 ± 48.52	161.64 ± 51.59	159.41 ± 52.74	0.003 *	0.938	0.053
Parafovea (µm)	Total retina	311.13 ± 24.33	284.98 ± 25.69	279.73 ± 22.45	297.41 ± 25.71	306.66 ± 24.48	306.42 ± 22.65	<0.001 *	<0.001 **	0.63
	RNFL	30.00 ± 9.31	29.36 ± 3.86	25.64 ± 10.12	31.61 ± 9.81	32.06 ± 11.14	28.10 ± 10.44	<0.001 *	<0.001 **	0.45
	GC-IPL	88.17 ± 14.15	80.92 ± 11.81	66.08 ± 15.35	78.47 ± 14.77	82.89 ± 17.96	84.58 ± 15.87	<0.001 *	<0.001 **	0.49
	Choroid	180.47 ± 41.89	173.23 ± 59.16	161.79 ± 44.16	174.95 ± 43.25	160.83 ± 46.93	159.20 ± 47.55	0.001 *	0.819	0.06
Optic disc (µm)	ppRNFL	110.65 ± 16.21	104.42 ± 17.29	92.0 ± 19.68	96.47 ± 18.54	96.58 ± 17.46	90.52 ± 15.35	0.026 *	0.290	0.003 ***

* Statistically significant difference (*p* < 0.05) by paired *t*-test between before SOR and 1 week after SOR, ** Statistically significant difference (*p* < 0.05) by paired *t*-test between before SOR and 3 months after SOR, *** Statistically significant difference (*p* < 0.05) by paired *t*-test between unaffected fellow eye and 6 months after SO removal. SOR, silicone oil removal; ETDRS, early treatment diabetic retinopathy study; RD, retinal detachment; SO, silicone oil; RNFL, retinal nerve fiber layer; GC-IPL, ganglion cell-inner plexiform layer; ppRNFL, peripapillary retinal nerve fiber layer.

**Table 3:** Linear mixed model of the retinal and choroidal thickness measured before and after silicone oil removal (one week, three months, and six months).

	Linear Mixed Model		Post Hoc Analysis Derived Using Bonferroni (*p*-Value)					
	F	*p*-Value	Before SOR vs. 1 Week after SOR	One Week after SOR vs. 3 Months after SOR	3 Months after SOR vs. 6 Months after SOR	Before SOR vs. 3 Months after SOR	One Week after SOR vs. 6 Months after SOR	Before SOR vs. 6 Months after SOR
**Fovea**								
Total retina	0.941	0.426	-	-	-	-	-	-
RNFL	0.043	0.988	-	-	-	-	-	-
GC-IPL	1.488	0.226	-	-	-	-	-	-
Choroid	0.164	0.920	-	-	-	-	-	-
**Parafovea**								
Total retina	2.731	0.049 *	0.673	1.0	1.0	0.139	1.0	0.048 ^†^
RNFL	4.231	0.009 *	0.054	1.0	0.516	0.009 ^†^	1.0	0.729
GC-IPL	5.137	0.003 *	0.123	1.0	1.0	0.012 ^†^	1.0	0.004 ^†^
Choroid	0.244	0.865	-	-	-			
**Optic disc** ppRNFL	0.335	0.80	-	-	-			

Bonferroni post hoc comparisons were conducted in the case of statistically significant changes. Post hoc analysis using the Bonferroni correction was performed. * Statistically significant difference (*p* < 0.05, Linear mixed model). ^†^ Statistically significant difference (*p* < 0.05, post hoc analysis derived using Bonferroni). SOR, silicone oil removal; SO, silicone oil; RNFL, retinal nerve fiber layer; GC-IPL, ganglion cell-inner plexiform layer; ppRNFL, peripapillary retinal nerve fiber layer.

**Table 4:** Comparison of each retinal thickness before retinal detachment surgery and 6 months after silicone oil removal in the parafoveal areas based on the maintenance period of silicone oil.

SO Tamponade Period	Area		Unaffected Fellow Eyes	6 Months after SO Removal	*p*-Value
≤3 months (*n* = 19)	Parafovea	Total retina (µm)	307.16 ± 18.14	295.65 ± 17.58	0.09
		RNFL (µm)	27.75 ± 9.32	26.97 ± 13.87	0.64
		GC-IPL (µm)	87.16 ± 16.75	84.25 ± 14.83	0.67
	Optic disc	ppRNFL	107.12 ± 19.75	95.77 ± 18.16	0.07
>3 months (*n* = 11)	Parafovea	Total retina (µm)	319.20 ± 20.55	303.59 ± 21.32	0.33
		RNFL (µm)	35.40 ± 10.80	30.82 ± 12.24	0.59
		GC-IPL (µm)	89.0 ± 17.10	85.4 ± 15.88	0.66
	Optic disc	ppRNFL (µm)	105.4 ± 24.28	79.0 ± 17.23	0.019 *

* Statistically significant difference (*p* < 0.05) by paired *t*-test between unaffected fellow eye and 6 months after SO removal. SO, silicone oil; RD, retinal detachment; RNFL, retinal nerve fiber layer; GC-IPL, ganglion cell-inner plexiform layer; ppRNFL, peripapillary retinal nerve fiber layer.

## Data Availability

The data that support the findings of this study are available from the corresponding author upon reasonable request.
